# Chicken or the leg: Sigmoid colon perforation by ingested poultry fibula proximal to an occult malignancy^☆^

**DOI:** 10.1016/j.ijscr.2013.08.001

**Published:** 2013-08-12

**Authors:** J.D. Terrace, J. Samuel, J.H. Robertson, R.G. Wilson, D.N. Anderson

**Affiliations:** Colorectal Unit, Western General Hospital, Crewe Road South, Edinburgh EH4 2XU, United Kingdom

**Keywords:** Bone perforation, Colon cancer, Radiology surgery

## Abstract

**INTRODUCTION:**

Colonic perforation by ingested foreign bodies is exceedingly rare, with the diagnosis made more challenging by patients infrequently recalling any inadvertent ingestion and the poor sensitivity of plain radiography.

**PRESENTATION OF CASE:**

The presented case demonstrates that bony perforation of the large bowel might occur immediately proximal to an otherwise occult colonic malignancy.

**DISCUSSION:**

Ingestion of foreign bodies is common and rarely results in colonic perforation. However, bony ingestion is not usually remembered and can be missed even with cross-sectional imaging. If present, consideration should be given to the presence of an adjacent concealed colon cancer.

**CONCLUSION:**

The co-existence of separate pathology should be carefully assessed in these patients, since this has important implications for relevant investigations and appropriate surgical management.

## 1 Introduction

Perforation of the colon, a surgical emergency encountered as a result of complicated diverticulitis, mechanical neoplastic obstruction or fulminant colitis, can be secondary to inserted or ingested foreign bodies. Food-derived bony items swallowed inadvertently can result in large bowel perforation but occasionally dual mechanisms of perforation co-exist and can represent a diagnostic challenge.

## 2 Case report

An 85-year-old male taking ferrous sulphate for anaemia, with previous peptic ulcer disease and ischaemic heart disease, presented as an emergency with 48 h of progressive of left-sided lower abdominal pain on a background of 2 months of loose stool. Admission physiological parameters were normal. There was tenderness in the left iliac fossa but the abdomen was soft and the rectum empty. Clinical examination was otherwise unremarkable.

Admission blood work confirmed anaemia with a haemoglobin of 98 g/l and elevated leucocyte and C-reactive protein counts of 11.6 × 10^9^ cells/l and 187 mg/l, respectively.

**Fig. 1:**
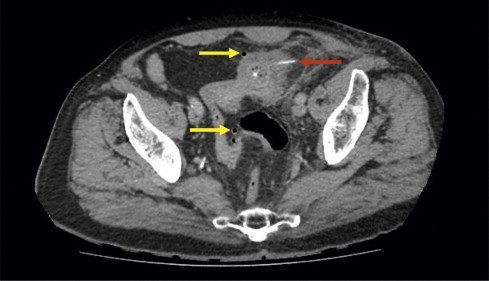
CT scan of abdomen and pelvis showing radio-dense bony foreign body perforating through the sigmoid colon (red arrow). Flecks of free intra-peritoneal air can be seen (yellow arrows).

**Fig. 2:**
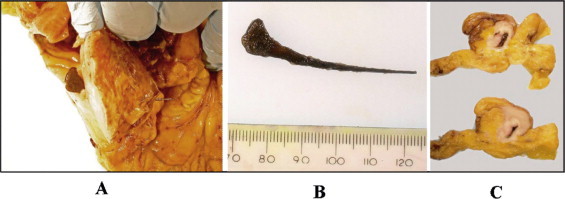
Pathology specimen images demonstrating perforation of a chicken bone through the sigmoid colon immediately proximal to a stenosing malignant lesion. A – chicken bone *in situ* with the broad end in the lumen and narrow end penetrating the serosal surface. B – chicken bone after removal. C – serial macroscopic sections of distal sigmoid colon showing luminal stenosing malignancy.

Plain abdominal X-rays were reviewed and the patient was admitted to the medical receiving unit for treatment of presumed constipation. The patient clinically decompensated with the development of increasingly severe abdominal pain and sepsis characterised by high fever and a tachycardia of 121 bpm. An erect chest X-ray to exclude a source for sepsis revealed pneumoperitoneum. An urgent surgical consultation identified an incarcerated, erythematous left-sided inguinal hernia and a CT scan of the abdomen and pelvis showed a mid-sigmoid perforation as a result of an ingested bone with a localised collection and free gas tracking through the left inguinal canal to the scrotum. Fluid resuscitation and appropriate antibiotic therapy was commenced and emergency laparotomy undertaken. This confirmed a perforation of the sigmoid colon by a chicken bone and also an immediately distal malignant lesion later characterised pathologically as a T3N1 adenocarcinoma. An anterior resection and colo-rectal anastomosis was performed, but the inguinal hernia was not repaired. The patient made a gradual but uneventful recovery and was discharged on the 15th postoperative day (Figs. 1 and 2).

## 3 Discussion

Although ingestion of foreign bodies is a commonly encountered clinical scenario thought to account for some 6000 presentations per year in the United Kingdom,^1^ the majority transit the gastro-intestinal tract spontaneously. Perforation remains a rare complication, occurring in less than 1% of patients,^1–3^ with the most frequent objects including toothpicks, fish bones, poultry bones and dentures.^2,4–9^ As a consequence of the infrequency of gut perforation and because patients rarely recall swallowing non-food objects, this condition is typically mis-diagnosed.^2,4,6^ In this case, the patient was initially admitted medically for treatment for constipation and urinary tract infection before the incidental finding of sub-diaphragmatic free gas. Moreover, following surgical consultation, a previously unrecognised incarcerated inguinal hernia was thought to be responsible for the patient's worsening clinical features and pneumoperitoneum. In previous cases, a plethora of presenting characteristics and subsequent investigations have been reported before the definitive diagnosis is reached.^5^ A narrow wooden or bony object renders plain radiography unhelpful and cross-sectional imaging in the form of CT scanning is necessary, although this modality can still overlook bony viscus perforation later identified at laparotomy or endoscopically.^4,5,9^

It has been suggested that those locations in the gut with tapering or angulation are most susceptible to perforation by foreign bodies.^3,4,7,9^ Accordingly, the most common sites include the terminal ileum and the recto-sigmoid junction. However, pre-existing pathological conditions might also make the intestinal wall more prone to penetration. Diverticulosis is the most common in the sigmoid colon and there have been previous instances of chicken bones protruding through a diverticulum, mimicking perforated diverticulitis^1,3–5^ or, where the sigmoid colon is unduly mobile, acute appendicitis.^6^ Conversely, ingested chicken bones have been identified as the cause of fistulating disease, presumably on a background of preceding diverticulosis.^3^ However, sigmoid cancer with luminal narrowing is not uncommon and the primary presentation may arise as a sequel to foreign body perforation of the colon.^7,9^ In this case, as with each of the 5 reported previously, an occult, locally advanced adenocarcinoma of the sigmoid colon immediately distal to the perforation was identified either at laparotomy or following pathological assessment. The cancer was not necessarily identified on pre-operative CT scanning. In 4 of these previous cases the cause was also an ingested poultry bone.^7^

## 4 Conclusion

Perforation of the gastro-intestinal tract by an ingested foreign body is a rare event, where meticulous clinical assessment is required to reduce diagnostic error and delay in surgical management. However, this is made all the more challenging by the typical absence of a corroborative history. Where foreign body perforation of the sigmoid colon has occurred, CT imaging should be carefully scrutinised for occult malignancy, as should a thorough examination of the colon distal to the perforation site. Obviously this finding will have implications for operative strategies.

## Conflict of interest statement

None declared.

## Funding

None.

## Ethical approval

Written informed consent was obtained from the patient for publication of this case report and accompanying images. A copy of the written consent is available for review by the Editor-in-Chief of this journal on request.

## Author contributions

J. Terrace: reviewed case, conceived manuscript structure, content and design, performed literature review, wrote manuscript, prepared figures.

J. Samuel: reviewed case, contributed to manuscript structure and writing.

J. Robertson: reviewed and edited manuscript and figures.

R.G. Wilson: management of the clinical case and reviewing the manuscript.

D. Anderson: reviewed, edited and co-wrote manuscript.
